# Editorial: Antimicrobial Resistance as a Global Public Health Problem: How Can We Address It?

**DOI:** 10.3389/fpubh.2020.612844

**Published:** 2020-11-12

**Authors:** Luciene Andrade Da Rocha Minarini, Leonardo Neves de Andrade, Eliana De Gregorio, Filipa Grosso, Thierry Naas, Raffaele Zarrilli, Ilana L. B. C. Camargo

**Affiliations:** ^1^Department of Pharmaceutical Sciences, Federal University of São Paulo, Diadema, Brazil; ^2^School of Pharmaceutical Sciences of Ribeirão Preto, University of São Paulo, Ribeirão Preto, Brazil; ^3^Department of Molecular Medicine and Medical Biotechnology, University of Naples Federico II, Naples, Italy; ^4^Faculty of Pharmacy, University of Porto, Porto, Portugal; ^5^Assistance Publique Hopitaux De Paris, Paris, France; ^6^Department of Public Health, University of Naples Federico II, Naples, Italy; ^7^Laboratory of Molecular Epidemiology and Microbiology (LEMiMo), São Carlos Institute of Physics, University of São Paulo, São Carlos, Brazil

**Keywords:** antimicrobial resistance, CRISPR-Cas, oxazolidinone-resistant *Enterococcus faecalis*, carbapenem-resistant *A. baumannii*, colistin-resistant *E. coli*, antibiotic-resistant non-typhoidal Salmonella, MRSA, VRE

Misuse of antibiotics in agriculture, food production, and especially among humans and animals is the predominant factor in the emergence and spread of antimicrobial resistance, which might further lead to deaths from infections worldwide (1). More specifically, in the European Union, attributable deaths due to antimicrobial-resistant microorganisms were estimated to be 33,110 per year (2). At the same time, it is now easier to isolate and characterize antimicrobial-resistant bacteria in clinical settings or the environment (1). In 2017, the WHO described the most critical multidrug-resistant bacteria for which novel therapeutics are urgently needed (3). Without surprise, they belonged to the already known group, ESKAPE (*Enterococcus faecium, Staphylococcus aureus, Klebsiella pneumoniae, Acinetobacter baumannii, Pseudomonas aeruginosa*, and *Enterobacter* spp.) (4), which causes most of the healthcare-associated infections nowadays.

On the top of the WHO list are the critical priorities: carbapenem-resistant *A. baumannii, P. aeruginosa*, and Enterobacteriaceae, plus ESBL-producing Enterobacteriaceae (3). As high priorities are vancomycin-resistant *E. faecium, Staphylococcus aureus* (methicillin-resistant, vancomycin-intermediate and resistant), clarithromycin-resistant *Helicobacter pylori*, fluoroquinolone-resistant *Campylobacter* spp., fluoroquinolone-resistant *Salmonellae, Neisseria gonorrhoeae* (cephalosporin-resistant, fluoroquinolone-resistant) (3). The third part of the WHO list ranked as medium priority consists of penicillin-non-susceptible *Streptococcus pneumoniae*, ampicillin-resistant *Haemophilus influenzae*, and fluoroquinolone-resistant *Shigella* spp. (3). In addition to the hospital (patients and healthcare workers) (5–8), bacteria of this list can also disseminate through the community (causing infections in humans or pets) (9–11), food chain (10, 12–14), and environment (15, 16).

Overuse of antibiotics prescribed to humans and animals plus the improper use in agriculture can select resistant bacteria that emerge after a spontaneous mutation or after acquiring resistance genes through horizontal transfer mediated by mobile genetic elements (17). This selection can occur in both the human and non-human gastrointestinal tracts as well as in contaminated environments. Once they become resistant to antibiotics, clonal bacterial cells are selected in the microbial population and disseminates its daughter cells. Alternatively, plasmids carrying resistance genes are transferred to other bacteria through conjugation or transformation (17). In Switzerland, a recent study on freshwater samples from rivers, inland canals, and streams showed environmental dissemination of high-risk carbapenemase-producing Enterobacteriaceae with plasmids composed of elements identical to those of resistance plasmids retrieved from clinical and veterinary isolates locally and worldwide (15). The authors concluded that these plasmids carrying carbapenemase genes replicate and evolve pollutants of river ecosystems and represent a threat to public health and environmental integrity (15). ESBL-producing Enterobacteriaceae were isolated from a Tunisian semi-industrial pilot plant with biological treatment (WWPP) and its receiving river with high rates of CTX-M-15 and high genetic diversity (16). Therefore, the water treatment and appropriate use of antibiotics may represent important infection control measures to avoid antimicrobial resistance spread in hospitals and communities (1, 18).

Bacteria may grow planktonically but most often form biofilms in nature or human body infections (19). These biofilms are common in the hospital environment, especially in polymeric clinical devices, such as catheters and cardiac pacemakers. The inhibition of biofilm growth is of great importance in infection control and treatment of healthcare-associated infections owing to their inherent tolerance and “resistance” to antimicrobial therapies (19). In addition, biofilms constitute a great challenge in clinical settings since there is no “gold standard” available to reveal the presence of microbial biofilms.

The prevention of bacterial resistance spread is a significant challenge among healthcare professionals, especially in hospital settings. Surveillance of clones and lineages spread in a hospital environment or among patients, and the knowledge of their susceptibility to antimicrobials are crucial data to initiate a proper empiric treatment of hospital-acquired infections and infection prevention and control.

In many cases, precision medicine is the key to therapy since different microbes are prone to different remedies. New antimicrobials might seem more effective and promising, but some traditional compounds, such as beta-lactamase or efflux pump inhibitors that could prevent enzymatic action on known antibiotics or efflux pump activity, respectively, are also beneficial.

This Research Topic harbors 48 published manuscripts, including original research, review, opinion, and brief research reports addressing these subjects. The 443 involved authors focused on tackling antimicrobial resistance in bacteria. According to Merlin, although the increasing occurrence of antibiotic resistance among bacteria due to the antimicrobial agent consumption has been recognized for decades, only recently, the seriousness of the situation has been considered by international, national, and local health agencies. Some recommendations were defined in order to educate and improve the practices of health professionals and consumers. Most recommendations proposed coordinated actions in public health and veterinary/agricultural domains, limiting the inappropriate exposure of bacteria to antibiotics, in order to delay the natural evolution toward resistance and its propagation in the downstream environment, in a One Health context.

The chapters of this Research Topic show that it is crucial to pay attention to antimicrobial resistance in microorganisms from wild animals, pets, livestock, food, and the environment, in addition to those isolated from humans, to understand how antimicrobial resistance spreads. Lin et al. used a genomic approach to study the horizontal transfer of colistin resistance between polluted rivers and wild birds. Metadata analysis of *mcr-1*- positive multidrug-resistant *E. coli* strains, which were isolated from the environment of egret habitat (polluted river) and egret feces, showed highly homologous *mcr-1*-bearing IncI2 plasmids among isolates from different regions along the East Asian-Australian Flyway, suggesting that migratory birds may mediate the intercontinental transportation of colistin resistance.

Mahmud et al. call the attention to the prevalence and characterization of MDR and ESBL-producing *E. coli* in drinking water samples collected from Rohingya camps, Bangladesh. The authors described that 66 out of 384 *E. coli* isolates in this study were ESBL-producers, of which 71% (47/66) were MDR. Sixty-four percent (42/66) of the ESBL-producing *E. coli* carried one to seven plasmids ranging from 1 to 103 MDa. Only large plasmids with antibiotic resistance properties were found transferrable via conjugation. This study shows that drinking water samples could foster exposure and dissemination of MDR, ESBL-producing, and pathogenic *E. coli* lineages, posing a health risk to the people residing in the densely populated camps of Bangladesh.

Chen et al. investigated the antimicrobial resistance trends and characteristics of ESBL-producing *E. coli* isolates from pets and whether this correlates with antibiotic usage in the clinic. The authors observed high rates of resistance to β-lactams and fluoroquinolones and resistance to >3 antibiotic classes. ESBL-encoding genes *bla*_CTX−M_ (*n* = 44, 34.65%) and plasmid-mediated quinolone resistance genes *qnrB* (*n* = 119, 93.70%) were most common in 36 PFGEs types and 28 different sequence types (STs), including ST405 (7, 15.9%), ST131 (3, 6.8%), ST73, ST101, ST372, and ST827 (2, 4.5% each). Additionally, *bla*_NDM−5_ was detected in three isolates (*n* = 3, 2.36%, two ST101 and one ST405), and *mcr-1* was also observed in three colistin-resistant *E. coli* (ST6316, ST405, and ST46).

García-Meniño et al. performed molecular characterization of 35 prevalent strains of colistin-resistant *E. coli* from swine enteric colibacillosis in Spain. The resistome analysis showed six different *mcr* variant genes. In seven of them, the authors observed an association with ESBL. The summative presence of mechanisms associated with high-level resistance to quinolone/fluoroquinolones and colistin could confer adaptive advantages to prevalent pig *E. coli* strains. The authors also reported the co-occurrence of double colistin-resistance mechanisms in a significant number of *E. coli* isolates.

Mukherjee et al. observed increasing frequencies of antibiotic-resistant non-typhoidal Salmonella (NTS) infections in Michigan, which is not part of the FoodNet surveillance system in the USA. A total of 198 isolates, from 2011 to 2014, belonged to 35 different serovars with the predominance of Enteritidis (36.9%), followed by Typhimurium (19.5%), and Newport (9.7%). A total of 30 (15.2%) NTS isolates were resistant to ≥1 antibiotic, and 15 (7.5%) were resistant to ≥3 antimicrobial classes. They demonstrated the importance of surveillance, resistance frequency monitoring, and identification of risk factors that can aid in the development of new prevention strategies.

Almeida et al. described the coexistence of oxazolidinone resistance genes *cfr* and *optrA* in a MDR *E. faecalis* isolate belonging to ST29, obtained from a healthy piglet in Brazil in the context of surveillance study. Whole-genome sequencing revealed that the *cfr* gene was in a transposon-like structure of 7,759 nucleotides flanked by IS*1216E* and capable of excising and circularizing, distinguishing it from known genetic contexts for *cfr* in *Enterococcus* spp., while *optrA* was in an Inc18 broad-host-range plasmid of >58 kb. Both genes were able to conjugate. This study highlights the need for monitoring the use of antibiotics in the Brazilian swine production system to control the spread and proliferation of antibiotic resistance.

This Research Topic also presents studies reporting the mechanisms of resistance and lineages of bacteria of clinical importance. Carbapenem-resistant *A. baumannii* (CRAB) often causes fatal infections among seriously ill patients. Wong et al. used whole-genome sequencing, gene expression studies, and enzyme kinetics analyses to investigate the underlying carbapenem resistance mechanisms in 14 clinical *A. baumannii*. A large majority of isolates belonged to the International Clone II (IC-II), ST208. CRAB harbored *bla*_OXA−23_/*bla*_OXA−72_, or overexpression of the *bla*_OXA−51_ gene upon IS*Aba1* insertion, and had strong carbapenem-degrading activities. Over-production of carbapenem-hydrolyzing-class D-ß-lactamases (CHDLs) is the key mechanism of carbapenem resistance in the isolates studied.

Fosfomycin exhibits significant antimicrobial activity against a broad spectrum of pathogens, including *S. aureus*. Although widely described in gram-negative bacteria, fosfomycin's resistance mechanism is rarely reported among gram-positive bacteria (Xu et al.). The molecular mechanisms of 11 fosfomycin-resistant *S. aureus* isolates from a Teaching hospital in China were determined. The authors showed that the mutations in the *uhpT, glpT*, and *murA* genes and the overexpression of the efflux pump gene *tet38* under a subinhibitory concentration of fosfomycin might play a major role in conferring fosfomycin resistance in these isolates. In contrast, none of the resistant strains carried the *fosA, fosB, fosC, fosD*, and *fosX* genes, indicating that these genes might not be the primary factors mediating the resistance of *S. aureus* against fosfomycin. Furthermore, MLST analysis identified ST5708, a novel sequence type of *S. aureus* (Xu et al.).

The possible transference of resistance genes among different species and the consequences that these genes may cause in the cells were covered in this Research Topic, for instance, in the study by Xiang et al., which described the presence of *bla*_NDM−1_ in 54 out of 1,735 carbapenem-non-susceptible strains, including *K. pneumoniae, A. baumannii*, and *E. coli*. After analyzing the *bla*_NDM−1_ gene and its genetic environment, the authors hypothesized that, due to the presence of the *ble* and *tnpA* genes, *bla*_NDM−1_ originates from *A. baumannii*, which is retained in *K. pneumoniae* over a long period by transposition of mobile elements.

Mobile genetic elements harboring resistance genes, in certain cases, may reduce bacterial fitness. In this Research Topic, Li et al. investigated the physiological function of the *mcr-1* gene, which encodes an LPS-modifying enzyme, in *E. coli* K-12. The removal of the *mcr-1* gene not only reduced resistance to colistin, but also increased cell viability under high osmotic stress conditions and led to increased resistance to hydrophobic antibiotics. Increased expression of *mcr-1* also resulted in a decreased growth rate and changed the cellular morphology of *E. coli* (Li et al.).

Sharma et al. used a proteomic approach to study the growth and fitness of a carbapenem-resistant NDM-4-producing *K. pneumoniae* clinical isolate under meropenem stress. The authors showed the downregulation of flagellar, fimbriae, and pili proteins by mass spectrometry and system biology analysis of networking pathways. Sharma et al. suggested that these proteins might be used as targets for the development of novel therapeutics against antimicrobial-resistant *K. pneumoniae* infections.

This Research Topic also brings the search and analysis of the CRISPR-Cas system, a microbial adaptive immune system involved in defense against different types of mobile genetic elements, in whole plasmid and chromosomal sequences of multidrug-resistant *K. pneumoniae* from GenBank (Kamruzzaman and Iredell). The authors found a putative CRISPR-Cas system in 44 plasmids from *Klebsiella* species and identified an identical system in three plasmids from other *Enterobacteriaceae*, with CRISPR spacers targeting different plasmids and chromosome sequences. Additionally, the authors showed that plasmid type IV CRISPR may depend on the chromosomal type I-E CRISPRs for their competence (Kamruzzaman and Iredell). Different aspects are involved in the acquisition and transference of resistance determinants, and the presence of CRISPR-Cas in plasmids just showed us we still have a lot to understand.

In this Research Topic, several manuscripts described the diversity of the most important pathogenic bacterial species and their susceptibility profiles. For instance, in India, the antibiotic susceptibility and typing of 109 *S. aureus* strains isolated from a variety of infections showed that all *S. aureus* isolates were multidrug-resistant, virulent, and diverse irrespective of sources and place of isolation being assigned to 46 *spa*-types, 33 STs and eight clonal complexes (Aggarwal et al.).

Ai et al. studied the prevalence, characterization, and drug resistance of *S. aureus* fecal carriage among pediatric patient feces in Southern China and found that the fecal carriage rates were 20.0% for *S. aureus* and 4.5% for methicillin-resistant *S. aureus* (MRSA). Among the 76 STs, the authors found 25 new STs, and the most prevalent were ST188, ST6, and ST15 for methicillin-sensitive *S. aureus* (MSSA) and ST59 and ST45 for MRSA. The authors described high rates of penicillin, erythromycin, and clindamycin resistance among the isolates. However, CC59 (ST338 and ST59) and CC45 exhibited different antibiotic resistance patterns. Ai et al. indicated an urgency for strengthening the surveillance programs in China that could contribute to *S. aureus* infection prevention and treatment.

Bispo et al. analyzed the population structure of ocular MRSA isolates using a combination of MLST analysis, SCC*mec* typing, and detection of the panton-valentine leukocidin (PVL) coding gene. The 68 isolates (2014–2016) belonged to 14 different sequence types (STs) that grouped within two predominant clonal complexes: CC8 (47.0%) and CC5 (41.2%). Although the isolates were associated with lineages with community and hospital origins, the authors suggested that the niche specialization could act as a driver for the community structure observed.

In Iran, Goudarzi et al. conducted a study from August 2013 to July 2018 to investigate the prevalence and molecular epidemiology of inducible macrolide-lincosamide streptogramin B (MLSB) resistance in *S. aureus*. The prevalence of inducible MLSB (iMLSB) *S. aureus* increased from 7.5 to 21.7% during the study period. The authors observed a replacement of the CC22, predominant in 2013–2014, by CC8 in 2017–2018. Goudarzi et al. described for the first time the temporal shifts of iMLSB *S. aureus* isolates in Iran that identify predominant clones and treatment options for iMLSB *S. aureus*–related infections.

Vasilakopoulou et al. described the prevalence and risk factors of antimicrobial resistance by studying the gastrointestinal carriage of vancomycin-resistant enterococci (VRE) and carbapenem-resistant gram-negative bacteria (CRGN) in the fecal flora of the inpatients of a tertiary university hospital in Greece. The authors found high prevalence rates for VRE and CRGN carriage. Prolonged hospitalization and age were independent risk factors for VRE carriage, while CRGN carriage was associated with an increased risk of acquiring a resistant pathogen, prolonged hospital stay, and increased mortality.

In South Korea, Yoon et al. showed that most (84.5%) of the *A. baumannii* blood isolates belonged to the international clonal lineage II (ICLII), and 89.5% of the isolates were either multidrug- or extensively drug-resistant. The patient's old age, the sequential organ failure assessment score, and causative *A. baumannii* ST191 belonging to ICLII were risk factors for 30-day mortality (Yoon et al.).

Ferrari et al. identified the circulation of three distinct genome clusters of carbapenem-resistant KPC-producing *K. pneumoniae* in an intensive care unit in a hospital in Northern Italy. The authors described two clusters, one linked to ST512 and one to ST258; the ST258 isolates were also colistin-resistant and responsible for a high number of clinical infections (Ferrari et al.).

Ferreira et al. characterized KPC-producing *Serratia marcescens* strains isolated during an outbreak involving patients hospitalized in the intensive care unit (ICU) and neonatal intensive-care-unit (NIUC) of a Brazilian tertiary hospital. All isolates carried KPC-carbapenemase (*bla*_KPC_) and extended-spectrum beta-lactamase *bla*_TEM_ genes. In addition to the MDR profiles exhibited by almost 25% of isolates, 98.2% of the isolates had the genes codifying to the pore-forming toxin (ShlA), phospholipase with hemolytic and cytolytic activities (PhlA), flagellar transcriptional regulator (FlhD), and positive regulator of prodigiosin and serratamolide production (PigP).

Due to increasing resistance to multiple antibiotics classes, the search for molecules displaying potent activity against multidrug-resistant (MDR) bacteria is mandatory. Additionally, the discovery of new therapeutic approaches and/or new pathways to be inhibited is an open door for a directed search for bioactive compounds. As an example, Barra et al. discussed the possibilities, using an *in silico*-based analysis, of enzyme inhibition of two new possible druggable biosynthesis pathways present in most bacteria, fungi, and plants but incomplete in humans: the thiamine (vitamin B1) and the pyridoxal 5′-phosphate and its vitamers (vitamin B6).

Loubet et al. discussed alternative therapies and preventative strategies for urinary tract infections caused by multidrug-resistant uropathogens, focusing on the following phases of the pathogenesis: colonization, adherence of pathogens to uroepithelial cell receptors, and invasion. In this review, the authors discussed vaccines, small compounds, nutraceuticals, immunomodulating agents, probiotics, and bacteriophages, highlighting the challenges each of these approaches face Loubet et al.

Zhang et al. investigated the effects of pharmacological doses of copper, an essential microelement for animals, on the microbial communities in the hindgut, and the antimicrobial resistance profiles of *E. coli* in weaned piglets. The pharmacological dose of copper affected the composition of the microbial community (affected the microbial metabolic functions of energy metabolism, protein metabolism, and amino acid biosynthesis), increased antimicrobial resistance (to chloramphenicol and ciprofloxacin) of intestinal *E. coli*, and was most likely harmful to the health of piglets at the early stage after weaning (Zhang et al.).

The new anti-biofilm or antimicrobial agents' analysis, as well as their mode of action, were assessed in some studies. Murugan et al. described that arborine and skimmianine compounds from ethyl acetate extract of *Gycosmis pentaphylla* harbor a high antibacterial activity against MDR *S. aureus*. Wang et al. showed that the flavone glycoside baicalin inhibits biofilm formation and the quorum-sensing system in *Staphylococcus saprophyticus*, thus suggesting that it might be a potential therapeutic agent for *S. saprophyticus* biofilm-associated infections.

She et al. described the potent antimicrobial activity of auranofin against MRSA/MSSA and *E. faecalis*, both planktonic cells and biofilms, with minimum inhibitory concentrations ranging from 0.125 to 0.5 mg/L. Auranofin, in combination with linezolid or fosfomycin, showed synergistic antimicrobial activities against MSSA and MRSA both *in vitro* and in a mouse infection model. There was also a synergistic effect with chloramphenicol against *E. faecalis*. Additionally, auranofin improved the antibiofilm efficacy of chloramphenicol and linezolid, even on biofilms grown on a catheter surface. Therefore, they showed that the combination of auranofin with linezolid, fosfomycin, and chloramphenicol provides a synergistic microbicidal effect *in vitro* and *in vivo*, which rapidly enhances antimicrobial activity.

Lytic bacteriophage therapy is a potential antibacterial therapy. Montso et al. characterized lytic *E. coli* O177-specific bacteriophages isolated from cattle feces. A total of 31 lytic *E. coli* O177-specific bacteriophages were isolated and 71% of these phage isolates produced large plaques on 0.3% soft agar. Selected phage isolates had a similar morphotype (an icosahedral head and a contractile tail) and were classified under the order Caudovirales, *Myoviridae* family. Montso et al. demonstrated that lytic *E. coli* O177-specific bacteriophages isolated from cattle feces are highly stable and can infect different *E. coli* strains.

Among the papers, the authors analyzed new antimicrobials and different compounds against MDR bacteria. Cefoselis, a fourth-generation cephalosporin, was evaluated by Cheng et al. against bacterial pathogens in China. The authors observed that cefoselis showed good activity against non-ESBL-producing *E. coli, K. pneumoniae*, and *P. mirabilis*, MSSA, and was also potent against Enterobacteriaceae, *P. aeruginosa*, and *Streptococcus*.

Omadacycline, a new tetracycline-class broad-spectrum aminomethylcycline, exhibited excellent *in vitro* activity against both MRSA and MSSA clinical isolates (Bai et al.). Omadacycline susceptibility in *S. aureus* may be affected by efflux pump proteins (i.e., a branched-chain amino acid transport system II carrier protein and a Na/Pi cotransporter family protein) (Bai et al.).

Zheng et al. compared the effects of radezolid and linezolid on planktonic and biofilm cells of *E. faecalis*. After analyzing 302 *E. faecalis*, they observed that the MIC50/MIC90 values of radezolid (0.25/0.50 mg/L) were eight-fold lower than those of linezolid (2/4 mg/L). They observed that the radezolid MICs against the high-level linezolid-resistant isolates (linezolid MICs ≥64 mg/L) increased to ≥4 mg/L with mutations in the four copies of the V domain of the 23S rRNA gene. In addition, they showed the involvement of OG1RF_12220 (mdlB2, multidrug ABC superfamily ATP-binding cassette transporter) in the alteration of radezolid and linezolid susceptibility. Zheng et al. described that Radezolid inhibited *E. faecalis* biofilm formation to a greater extent than linezolid. Therefore, radezolid is more effective than linezolid against *E. faecalis*.

Hao et al. showed a potent *in vitro* activity of apramycin, an antibiotic with good activity against a range of multidrug-resistant pathogens, against clinical carbapenem-resistant, and hypervirulent *K. pneumoniae* (CR-hvKp) along with carbapenem-resistant non-hvKp (CR-non-hvKp) isolates, including those resistant to amikacin or gentamicin.

Dong et al. demonstrated that ebselen, an organo-selenium with well-characterized toxicology and pharmacology, has high bactericidal activity against multidrug-resistant (MDR) *S. aureus* based on taking TrxR as a major target and disruption of the redox microenvironment. The authors showed that ebselen is an effective topical antibacterial agent in animal model of MDR *S. aureus* LT-1 skin infection, reducing the bacterial load and the expression of pro-inflammatory cytokines.

Murugan et al. focused on the isolation and structural characterization of bioactive compounds from the ethyl acetate extract of *Gycosmis pentaphylla*. The isolated compounds identified as arborine and skimmianine have high bactericidal effects against MDR *S. aureus* clinical isolates. These compounds induced intracellular molecular imbalance and cell membrane disturbances that caused cell death of all MDR *S. aureus* strains and the reference standard *S. aureus* MTCC-96 strain.

A bottleneck in clinical microbiology, the incubation time necessary to the susceptibility test is ready for the antibiotic decision by the clinician is something to overcome. Genomics and technology can work together to improve the turnaround time in the clinical laboratory. Liu et al. used 2 genotype-based machine learning methods named Support Vector Machine (SVM) and Set Covering Machine (SCM) to predict the resistance to tetracycline, ampicillin, sulfisoxazole, trimethoprim, and enrofloxacin. The correlation results between the phenotype and the model predictions of the five antimicrobial agents indicated that both SVM and SCM models could significantly discern the resistant isolates of the sensitive isolates and could be potential tools in antimicrobial resistance surveillance and clinical diagnosis in veterinary medicine.

Resistance mechanisms, surveillance of antimicrobial-resistant bacteria, and new drug discovery studies offer different views of the antimicrobial resistance, which became a public health problem. There are more articles dedicated to the community focused on antimicrobial resistance in this Research Topic, and we hope you enjoy it.

## Author Contributions

All authors wrote about the manuscripts they edited in the Research Topic. IC also compiled and edited the complete text.

## Conflict of Interest

The authors declare that the research was conducted in the absence of any commercial or financial relationships that could be construed as a potential conflict of interest.
